# Oral Route Driven Acute *Trypanosoma cruzi* Infection Unravels an IL-6 Dependent Hemostatic Derangement

**DOI:** 10.3389/fimmu.2019.01073

**Published:** 2019-05-14

**Authors:** Dina Antunes, Alessandro Marins-Dos-Santos, Mariana Tavares Ramos, Barbara Angelica S. Mascarenhas, Carlos José de Carvalho Moreira, Désio Aurélio Farias-de-Oliveira, Wilson Savino, Robson Q. Monteiro, Juliana de Meis

**Affiliations:** ^1^Laboratory on Thymus Research, Oswaldo Cruz Institute, Oswaldo Cruz Foundation, Rio de Janeiro, Brazil; ^2^National Institute of Science and Technology on Neuroimmunomodulation, Oswaldo Cruz Institute, Oswaldo Cruz Foundation, Rio de Janeiro, Brazil; ^3^Parasitic Diseases Laboratory, Oswaldo Cruz Institute, Oswaldo Cruz Foundation, Rio de Janeiro, Brazil; ^4^Institute of Medical Biochemistry Leopoldo de Meis, Federal University of Rio de Janeiro, Rio de Janeiro, Brazil

**Keywords:** Chagas disease, oral transmission, cytokine, platelet, coagulation, interleukin-6

## Abstract

Oral transmission of *Trypanosoma cruzi*, the etiologic agent of Chagas disease, is presently the most important route of infection in Brazilian Amazon. Other South American countries have also reported outbreaks of acute Chagas disease associated with food consumption. A conspicuous feature of this route of transmission is presenting symptoms such as facial and lower limbs edema, in some cases bleeding manifestations and risk of thromboembolism are evident. Notwithstanding, studies that address this route of infection are largely lacking regarding its pathogenesis and, more specifically, the crosstalk between immune and hemostatic systems. Here, BALB/c mice were orally infected with metacyclic trypomastigotes of *T. cruzi* Tulahuén strain and used to evaluate the cytokine response, primary and secondary hemostasis during acute *T. cruzi* infection. When compared with control uninfected animals, orally infected mice presented higher pro-inflammatory cytokine (TNF-α, IFN-γ, and IL-6) serum levels. The highest concentrations were obtained concomitantly to the increase of parasitemia, between 14 and 28 days post-infection (dpi). Blood counts in the oral infected group revealed concomitant leukocytosis and thrombocytopenia, the latter resulting in increased bleeding at 21 dpi. Hematological changes paralleled with prolonged activated partial thromboplastin time, Factor VIII consumption and increased D-dimer levels, suggest that oral *T. cruzi* infection relies on disseminated intravascular coagulation. Remarkably, blockade of the IL-6 receptor blunted hematological abnormalities, revealing a critical role of IL-6 in the course of oral infection. These results unravel that acute *T. cruzi* oral infection results in significant alterations in the hemostatic system and indicates the relevance of the crosstalk between inflammation and hemostasis in this parasitic disease.

## Introduction

The hemoflagellate protozoan *Trypanosoma cruzi* is the causative agent of Chagas disease or American trypanosomiasis which is transmitted primarily through contact with feces of triatomine insects after biting (1). Transmission can also occur through blood transfusion (2), organ transplantation (3), congenitally (vertical transmission) (4), laboratory accidents (5) and by ingestion of contaminated food/juices (6, 7). Currently, oral infection is the most frequent route of transmission in Brazil and other Latin American countries (7–9). Mortality rates in these orally infected patients are higher (8–35%) when compared with the classical vectorial transmission (<5–10%) (10). The acute phase of the disease is a critical period often accompanied by non-specific clinical symptoms, such as fever, asthenia, face and limb edema, headache, myalgia, and others. Minor bleeding manifestations, most commonly from nose, skin petechiae, or bruising, are apparent in some patients with oral acute Chagas disease (ACD) and occasionally, risk of thromboembolism is reported and digestive bleeding may cause death (7, 11–13). These clinical/hematological signs have a frequency of 4.9% in orally-transmitted ACD outbreaks (14), although higher values were described in some cases (12, 13). Moreover, the association between anemia and thrombocytopenia in the ACD was already envisioned by Carlos Chagas in 1909 (1).

The knowledge of immunological events that occur during ACD are mainly based on studies using murine models. *Trypanosoma cruzi* experimental infection leads to pathogen-associated molecular patterns (PAMPs) activation in macrophages and dendritic cells with IL-12 secretion. Furthermore, synthesis of interferon-γ (IFN-γ), tumor necrosis factor-α (TNF-α), and nitric oxide (NO) by macrophages contributes to parasite clearance (15). In a mouse model of *T. cruzi* oral infection with the Tulahuén strain, it was shown that the major source of TNF in infected tissues are macrophages and high levels of this cytokine are associated to cardiac, hepatic and spleen injuries as well as toxic shock in infected BALB/c (16, 17). IL-6 is also involved in host protective response since IL-6-/- mice presented 3-fold higher parasitemia and died earlier than wild-type *T. cruzi* infected animals, by the subcutaneous route (18). Interestingly, Th1 cytokines are involved in an intense crosstalk between immune and hemostatic systems. Acute inflammation, as a response of an infection can modulate the systemic activation of the coagulation cascade and impair physiological anticoagulant pathways (19–22).

Tissue factor (TF), the integral transmembrane protein that initiates coagulation, is strongly induced by pro-inflammatory cytokines and C reactive protein on monocytes, fibroblasts and endothelial cells surface allowing further interaction with factor VII to form the complex TF-factor-VIIa, ultimately resulting in fibrin formation (19, 23). Under normal conditions, cells in direct contact with circulating blood do not express physiologically active TF (24). The traditional coagulation cascade includes intrinsic and extrinsic pathways that lead to the activation of different coagulation factors converging at the activation of factor X to factor Xa. Factor Xa forms a complex with factor Va to activate prothrombin into thrombin. Thrombin then converts fibrinogen to a fibrin network forming the clot (22).

Baboons lethally challenged with *Escherichia coli* and infused with recombinant antithrombin (protease inhibitor of thrombin and factor Xa) at high concentrations, had lower IL-6 and IL-8 plasma levels and the mortality was markedly reduced (25). Furthermore, blockade of IL-6 with a monoclonal antibody, in a primate model of sepsis, attenuated the LPS-induced coagulation (26). This effect was independent of TNF, since abrogation of this cytokine with recombinant TNF receptor IgG fusion protein or a neutralizing TNF antibody in healthy humans or LPS injected chimpanzees had no effect in coagulation activation (26, 27). To the best of our knowledge, there are no studies focusing on the inflammatory and hematological crosstalk as well as their mechanisms in oral ACD. The few studies addressing this interaction in Chagas disease in literature focus on chronic *T. cruzi* infection and have controversial results regarding the existence of a prothrombotic status in *T. cruzi*-infected patients (28–30).

Here, by using a pre-established mouse model of oral ACD, we demonstrate that infection leads to a decrease in platelet count, increased bleeding and coagulation time, mainly in the peak of parasitemia. Importantly, circulating IL-6 levels seem to be involved in these hematological changes during oral *T. cruzi* infection. This information may help elucidating the mechanism of oral ACD pathogenesis and provide an additional view on the interaction between inflammation and coagulation in the context of infectious diseases.

## Materials and Methods

### Animals and Infection

Male BALB/c mice were obtained from ICTB Oswaldo Cruz Foundation animal facilities (Brazil) and maintained in SPF conditions. Mice (6–8 weeks old) were infected via oral cavity by pipetting 50 all of excreta into their mouth with 5 × 10^4^ *T. cruzi* insect-vector (*Triatoma infestans*)-derived metacyclic forms (Tulahuén strain, TcVI). A different group of mice received the same number of trypomastigotes by the subcutaneous route (SC) after a single inoculation in the dorsal region. Before the infection, mice were maintained starving 4 h and at least 15 min after inoculation.

### Ethics Statement

This study was performed in strict accordance with the recommendations in the Guide for the Care and Use of Laboratory Animals of the Brazilian National Council of Animal Experimentation and the Federal Law 11.794 (10/2008). The Institutional Ethics Committee for Animal Research of the Oswaldo Cruz Foundation (CEUA-FIOCRUZ, License: L-028/2016) approved all the procedures used in this study.

### Parasitemia

Parasitemia was detected at different dpi by counting trypomastigotes in 5 μL of tail blood and parasite number was calculated using the Pizzi-Brener method.

### Cytometric Bead Array (CBA)

Mice were anesthetized, bled by cardiac puncture and euthanized by exsanguination at 3, 7, 14, 21, 24, and 28 dpi. Each bleeding point represents one mouse. To measure IL-6, IFN-γ, and TNF-α cytokines in the serum, we used the BD Mouse Th1/Th2/Th17 Cytokine CBA kit (BD Biosciences, USA). The assays were performed according to manufacturer's instructions. Sera were stored frozen at −70°C until used.

### Blood Cell Analysis

The mice were anesthetized and blood was collected into EDTA BD microteiner® tube by cardiac puncture. Blood cell count was automatically determined using the Poch 100- iV DIFF hematology analyzer (Sysmex, Japan).

### Clotting Assays and Measurements of Coagulation Factors

Activated Partial Thromboplastin Time (aPTT) and Prothrombin time (PT) were evaluated on a STart 4 stagocoagulometer (DiagnosticaStago, USA). For the aPTT, plasma (50 μL) was incubated in the coagulometer for 5 min at 37°C. Then, 50 μL of pre-warmed aPTT reagent (STA PTT; DiagnosticaStago, France) was added and further incubated for 2 min CaCl_2_ (50 μL at 25 mM) was added to start reactions. For determining the PT, plasma (50 μL) was incubated in the coagulometer for 5 min at 37°C. Then, 100 μL of the PT reagent (NEOplastine CI plus; DiagnosticaStago, France) was added. Time for clot formation was recorded in duplicates.

Serum levels of coagulation factors (FV, FVII, FVIII, and APC) and D-dimer were determined using commercial enzyme linked immunoabsorbent assay (ELISA) kits according to manufacturer's protocol (ElabScience Biotechnology, China).

### Tail Bleeding Assay

Bleeding propensity was evaluated as previously described (31). Mice were anesthetized with intramuscular xylazin (16 mg/kg) and ketamine (100 mg/kg). After 15 min, the distal 2 mm segment of the tail was removed and immediately immersed in 40 mL distilled water warmed to 37°C during exactly 30 min. The samples were properly homogenized and the absorbance was determined at 540 nm in order to estimate the hemoglobin content.

### Anti-IL6R and Anti-TNF Treatment

Orally infected BALB/c mice were treated intraperitoneally with a monoclonal antibody against the interleukin-6 receptor (IL-6R) (8 mg/kg, Tocilizumab, Actemra®, Roche, Switzerland), IgG control antibody (8 mg/kg, chrompure IgG Jackson Immunoresearch Labs, USA) or with a chimeric anti-TNF protein (0.83 mg/Kg, Etanercept Enbrel®, Wyeth Pharmaceuticals, USA). The treatment began at the 14 dpi with 48 h subsequent doses in the case of anti-IL-6R antibody or IgG control antibody and with another dose at 18 dpi for the anti-TNF reagent. The control group received normal saline solution at the same volume (100 μL) and frequency as described for orally infected mice.

### Statistical Analysis

Data were subjected to the D'Agostino-Pearson normality test to determine whether they were sampled from a Gaussian distribution. If a Gaussian model of sampling was fulfilled, parametric test (one-way ANOVA with Tukey's multiple comparison test) was used. If the samples deviated from a Gaussian distribution, non-parametric test (Kruskal–Wallis with Dunn's multiple comparison test) was applied. All statistical analysis was done in GraphPad Prism 6 (GraphPad Software Inc.). *P* < 0.05 were considered statistically significant.

## Results

### Increased Pro-inflammatory Cytokine Secretion Parallels Parasitemia Elevation in Acute *T. cruzi* Oral Infection

**Figure 1:**
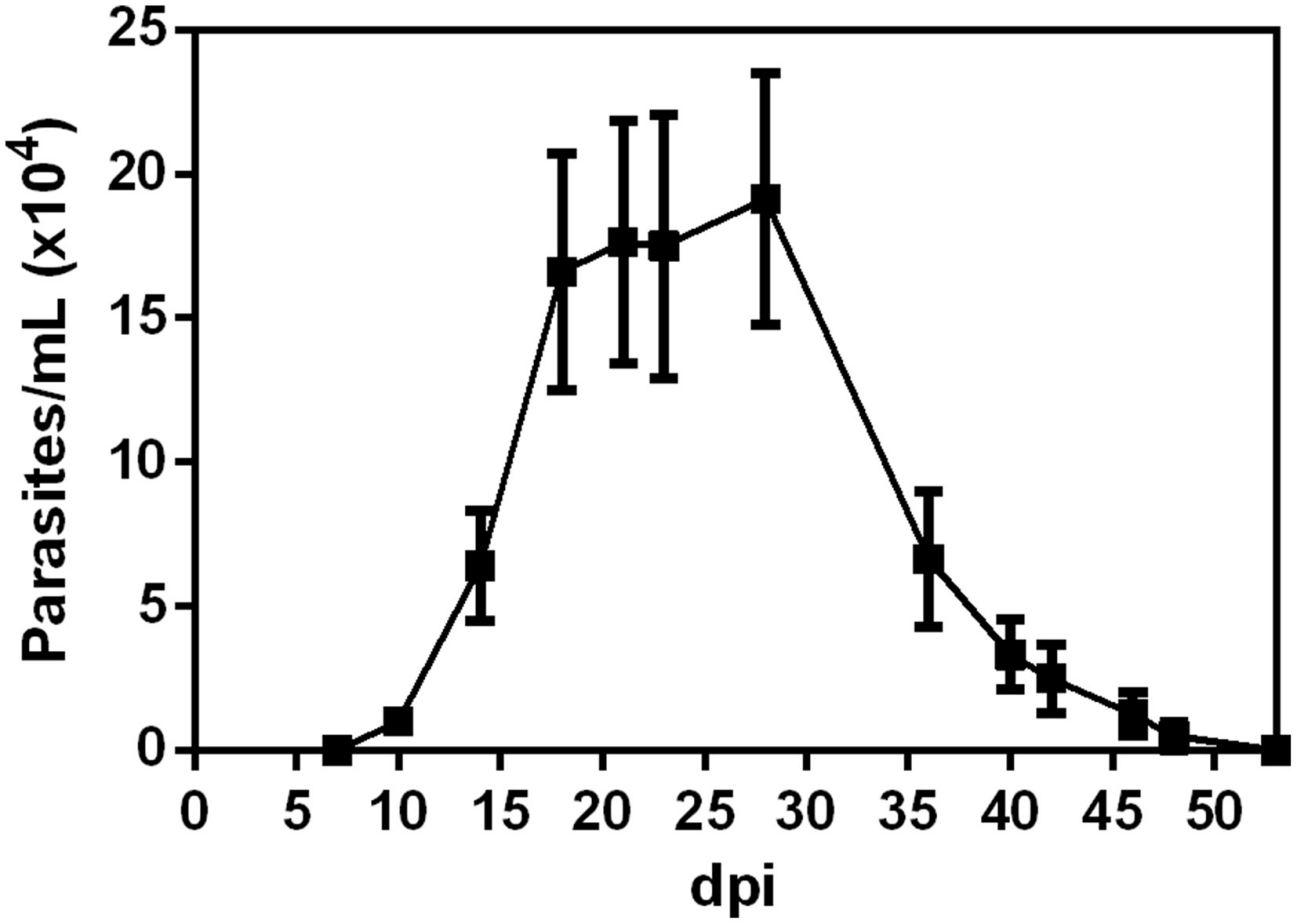
Parasitemia development. Male BALB/c mice were infected with 5 × 10^4^ insect-derived metacyclic forms of *T. cruzi* within the oral cavity. Parasitemia (mean and SEM) was assessed during the acute phase and is expressed as parasites per milliliter. Parasites were counted by light microscopy and parasitemia calculated by the Pizzi-Brener method. n: 7 dpi = 21, 10, and 14 dpi = 15, 18 dpi = 18, 21 dpi = 11, 23 dpi = 6, 28 dpi = 8; 36, 40, 42, 46, 48, and 53 = 4. The total number of animals in each time point was obtained from different experiments.

**Figure 2:**
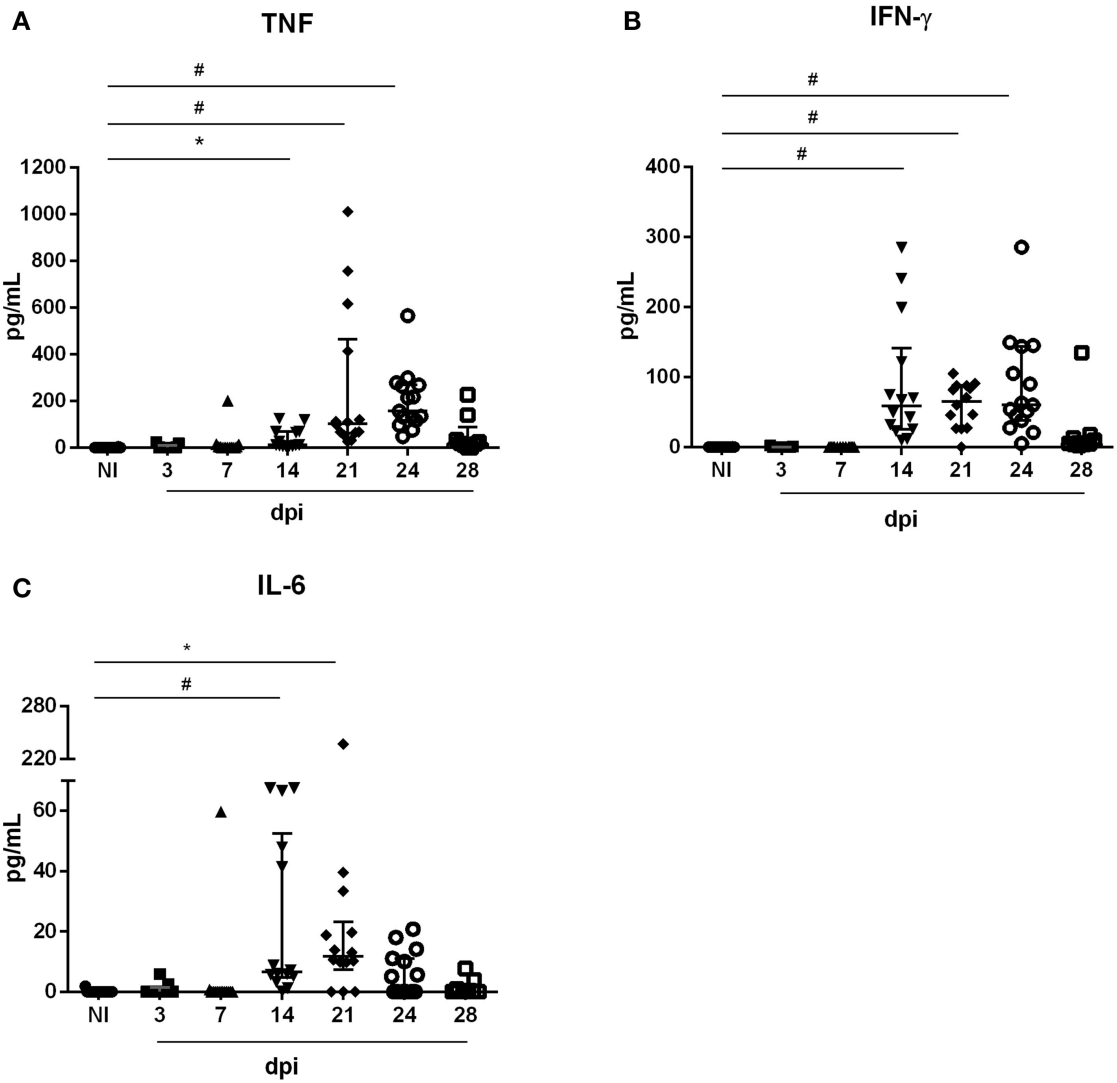
Serum cytokine levels during oral acute *T. cruzi* infection. Male BALB/c mice were infected with 5 × 10^4^ insect-derived metacyclic forms of *T. cruzi* within the oral cavity. In the course of the acute infection, serum was isolated and levels of TNF **(A)**, IFN-γ **(B)**, and IL-6 **(C)** were quantified in non-infected (NI) and infected mice by the CBA method. Values represent the median with interquartile range for each group/day post-infection and are representative of two independent experiments. Results were analyzed using Kruskal–Wallis with Dunn's multiple comparisons test (*0.0001< *p* < 0.05, #*p* < 0.0001). Significant differences not displayed in the graph: TNF: 3, 7 dpi ≠ 21, 24 dpi; IFN-γ: 3, 7 dpi ≠ 14, 21, 24 dpi; IL-6: 3 dpi ≠ 14 dpi e 7 dpi ≠ 14, 21 dpi. n: NI = 16; 3 e 28 dpi = 9; 7, 14 e 21 dpi = 14; 24 dpi = 15. n: NI = 16; 3 and 28 dpi = 9; 7, 14, and 21 dpi = 14; 24 dpi = 15.

We initially determined the parasitemia of BALB/c mice orally infected (OI) with 5 × 10^4^ *T. cruzi* metacyclic trypomastigotes. As shown in Figure 1, circulating parasites were first detected at 10 dpi. Furthermore, the animals showed higher numbers of parasites between 21 and 28 dpi. We next evaluated if *T. cruzi* infection caused transient changes in serum concentrations of pro-inflammatory cytokines. Accordingly, OI animals presented increased levels of TNF (Figure 2A), IFN-γ (Figure 2B) and IL-6 (Figure 2C) when compared with non-infected animals (NI). During 14–24 dpi, all cytokines exhibited high levels concomitantly to the increased numbers of circulating parasites (Figure 1). In contrast, there were no statistically significant differences in concentrations of TNF, IFN-γ, and IL-6 in the initial stages of infection (3–7 dpi), when parasitemia is not detected. Statistically significant differences in the Th2 and Th17 cytokine levels were not detected (Figure S1).

### Mice Orally Infected With *T. cruzi* Exhibit Signs of Disseminated Intravascular Coagulation, Including Thrombocytopenia and Increased Bleeding

We analyzed several hematological parameters in NI and OI mice on 7, 14, 21, and 28 dpi. As shown in Table 1, OI induced thrombocytopenia. On day 14 and 21, the platelet counts were 775.4 (±62.54) × 10^3^/μL and 840.8 (± 83.74) × 10^3^/μL for infected mice, respectively. There were no significant changes in red blood cell count, hemoglobin concentration, hematocrit and mean corpuscular volume when compared to NI. In addition, orally infected mice exhibited leukocytosis on 21 and 28 dpi (Table 1).

**Table 1:** Blood counts of non-infected (NI) and orally *T. cruzi* infected (OI) mice.

	**NI**	**7**	**14**	**21**	**28**
WBC	6.6 ± 0.86	9.1 ± 0.41	7.3 ± 0.60	16 ± 2.1^*^	18 ± 1.4^*^
RBC	9.8 ± 0.27	11 ± 0.20	9.5 ± 0.21	9.1 ± 0.27	10 ± 0.46
HGB	14 ± 0.33	16 ± 0.35	14 ± 0.41	13 ± 0.45	13 ± 0.49
MCV	52 ± 0.97	52 ± 0.42	49 ± 0.47	53 ± 0.35	49 ± 0.69
HMT	51 ± 0.58	58 ± 1.1	47 ± 1.3	48 ± 1.6	49 ± 1.6
PLT	1195 ± 81.71	1313 ± 101.0	775.4 ± 62.54^*^	840.8 ± 83.74^*^	1282 ± 17.44

Blood cells were measured on the Poch 100- iV DIFF hematology analyzer. Values are presented as mean ± SEM for each group/day post-infection and are representative of two independent experiments. Results were analyzed using the Kruskal-Wallis with Dunn's multiple comparisons test (

* *≠ NI and 0.0001 < p < 0.05. Significant differences not displayed in the graph: WBC: 7, 14 dpi ≠ 21, 28 dpi; RBC: 7 dpi ≠ 21 dpi; HGB: 7 dpi ≠ 21, 28 dpi; MCV: 14 dpi ≠ 21, 28 dpi; HMT: 7 dpi ≠ 14, 21 dpi. n = 5). HGB, total hemoglobin (g/dL); HMT, hematocrit; MCV, mean corpuscular volume (fL); PLT, number of platelets (10^3^ cells/μL); RBC, number of red blood cells (10^6^ cells/μL); WBC, number of white blood cells (10^3^ cells/μL)*.

**Figure 3:**
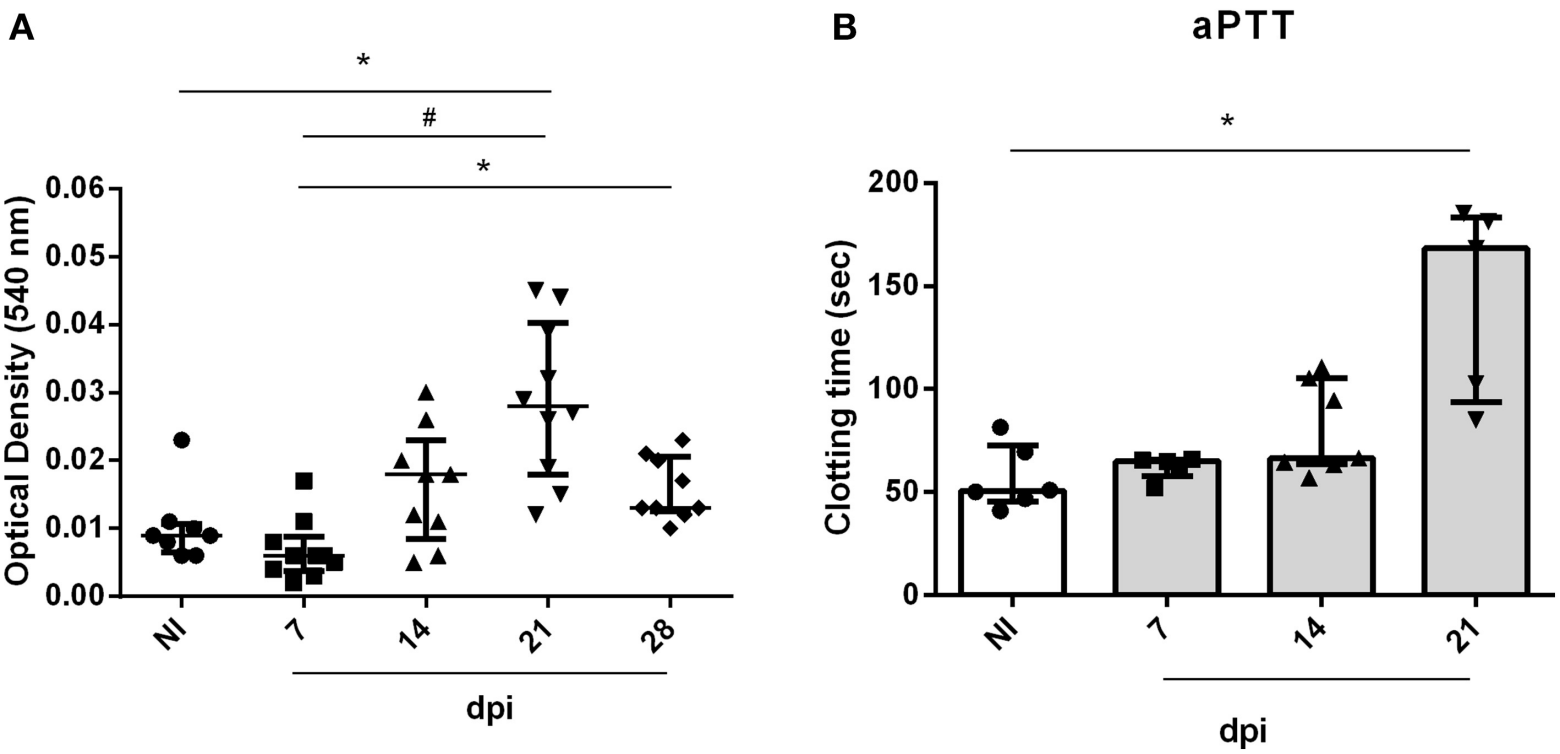
Bleeding from the tail of BALB/c mice and *ex vivo* PT and aPTT measurements during oral infection. Male BALB/c mice were infected with 5 × 10^4^ insect-derived metacyclic forms of *T. cruzi* (Tulahuén strain) within oral cavity. **(A)** Bleeding was caused by a tail transection in NI, 7, 14, and 21 dpi OI mice. Absorbance at 540 nm (hemoglobin concentration) was used to estimate blood loss. **(B)** NI or OI plasma samples were obtained by cardiac puncture followed by addition of aPTT or PT reagent as described in the “Methods” section. Clotting time was estimated using a coagulometer. **(A,B)** Values represent the median with interquartile range for each group/day post-infection and are representative of two independent experiments. Results were analyzed using the Kruskal-Wallis with Dunn's multiple comparisons test (*0.0001< *p* < 0.05, #*p* < 0.0001).

In order to evaluate the impact of acute infection-induced thrombocytopenia, the tail transection method was employed to evaluate the bleeding tendency. As seen in Figure 3A, OI mice showed a marked increase in bleeding at 21 dpi when compared with the NI counterparts.

The elevated pro-inflammatory cytokine profile concomitant with the decrease in platelet count suggests that oral infection may course with disseminated intravascular coagulation. In order to test this hypothesis, plasma from mice were collected and used for determination of *ex vivo* aPTT and PT coagulation tests. Oral infection with *T. cruzi* prolonged the aPTT (Figure 3B) but did not affect the PT (data not show). This is compatible with a derangement in the intrinsic pathway of the coagulation cascade.

Comparison of the coagulation factor levels between NI and OI showed statistically significant differences for FV, FVII, and FVIII (Figure S2) with a consumption of FVIII at 14 dpi. D-Dimer, a fibrin degradation product that marks fibrin generation, degradation and reflects the turnover of the coagulation system, was also measured in NI and OI. The concentration of this biomarker was detected on 14 and 21 dpi in OI (Table 2).

**Table 2:** Serum contents of D-dimer during acute phase of oral *T. cruzi* infection.

**Group**	**Mice with D-dimer>0 ng/mL**
NI	0/6
7 dpi	0/5
14 dpi	3/6
21 dpi	2/6

*Male BALB/c mice were infected with 5 × 10^4^ insect-derived metacyclic forms of T. cruzi within the oral cavity. NI or OI sera were obtained by cardiac puncture and used to measure levels of D-dimer by ELISA. The mean and SEM of animals with detectable levels of D-dimer were 25.7 ± 7.59 and 45.1 ± 11.0 for 14 and 21 dpi, respectively*.

### Blocking of IL-6 Signaling Prevents Hematological Changes in the Murine Model of *T. cruzi* Oral Infection

**Figure 4:**
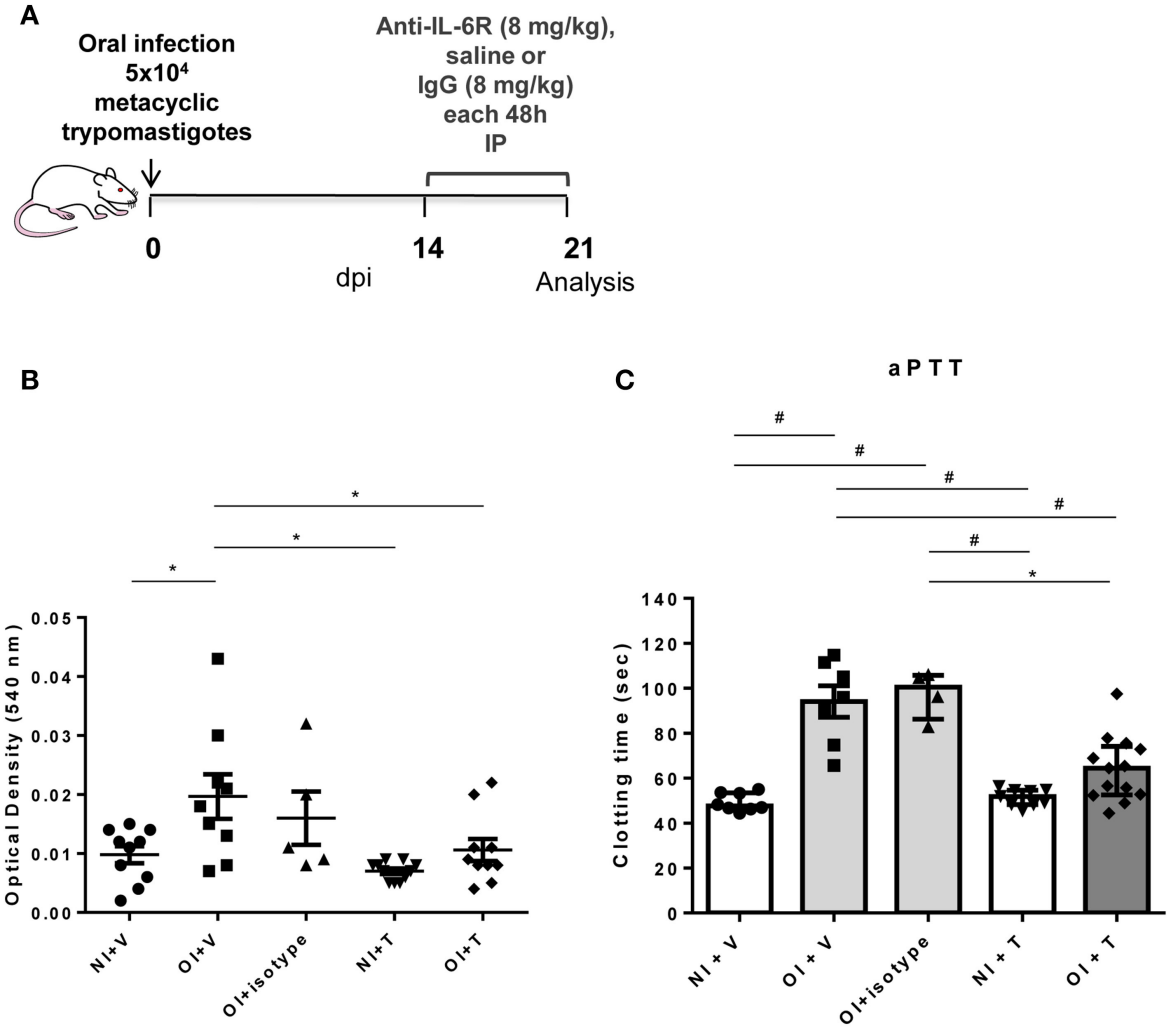
Experimental design, coagulation test aPTT and tail bleeding assay in NI+V, OI+V, OI+isotype, NI+T and OI+T mice. **(A)** Male BALB/c mice were infected with 5 × 10^4^ insect-derived metacyclic forms of *T. cruzi* (Tulahuén strain) within the oral cavity. Anti-IL6R or IgG treatment began after 14 dpi and was performed in each 48 h. **(B)** Bleeding was caused by a tail transection in NI+Vehicle (NI+V), OI+V, OI+isotype, NI+treatment (NI+T) and OI+T. Absorbance at 540 nm (hemoglobin concentration) was used to estimate blood loss. **(C)** NI+V, OI+V, OI+isotype, NI+T and OI+T plasma were obtained by cardiac puncture followed by addition of the aPTT reagent as described in the “Methods” section. Clotting time was estimated using a coagulometer. **(B,C)** Values are presented as mean ± SEM for each group/day post-infection and are representative of three independent experiments. Results were analyzed using one way ANOVA with Tukey's multiple comparisons test (*0.0001< *p* < 0.05; #*p* < 0.0001).

Oral infected mice had higher pro-inflammatory cytokine serum levels, especially between 14 and 24 dpi (Figure 2). Therefore, we hypothesized that blockade of IL-6R or soluble TNF could attenuate hemostatic changes observed in OI mice. To test this hypothesis, we treated OI mice with Tocilizumab (T), a monoclonal antibody that targets IL-6 signaling by competing for IL-6R (Figure 4A) or with a quimeric anti-TNF protein, etanercept (Enbrel®) (Figure S3A). Both treatments started at 14 dpi. Treated OI mice group (OI+T) had a significant drop in bleeding compared with vehicle alone (saline) OI+V (Figure 4B). Moreover, OI+T had lower aPTT than OI+V and OI+isotype and showed no significant differences between non-infected controls (NI+V or NI+T) (Figure 4C). In contrast, values recorded for aPTT and bleeding assays remained unaltered after anti-TNF treatment (Figures S3B,C). These results suggest that blocking the IL-6R attenuates changes in the hemostatic system under *T. cruzi* oral infection whereas TNF blockade did not influence those alterations.

### Hematological Disturbances Are Also Observed in Subcutaneously Infected Mice

**Figure 5:**
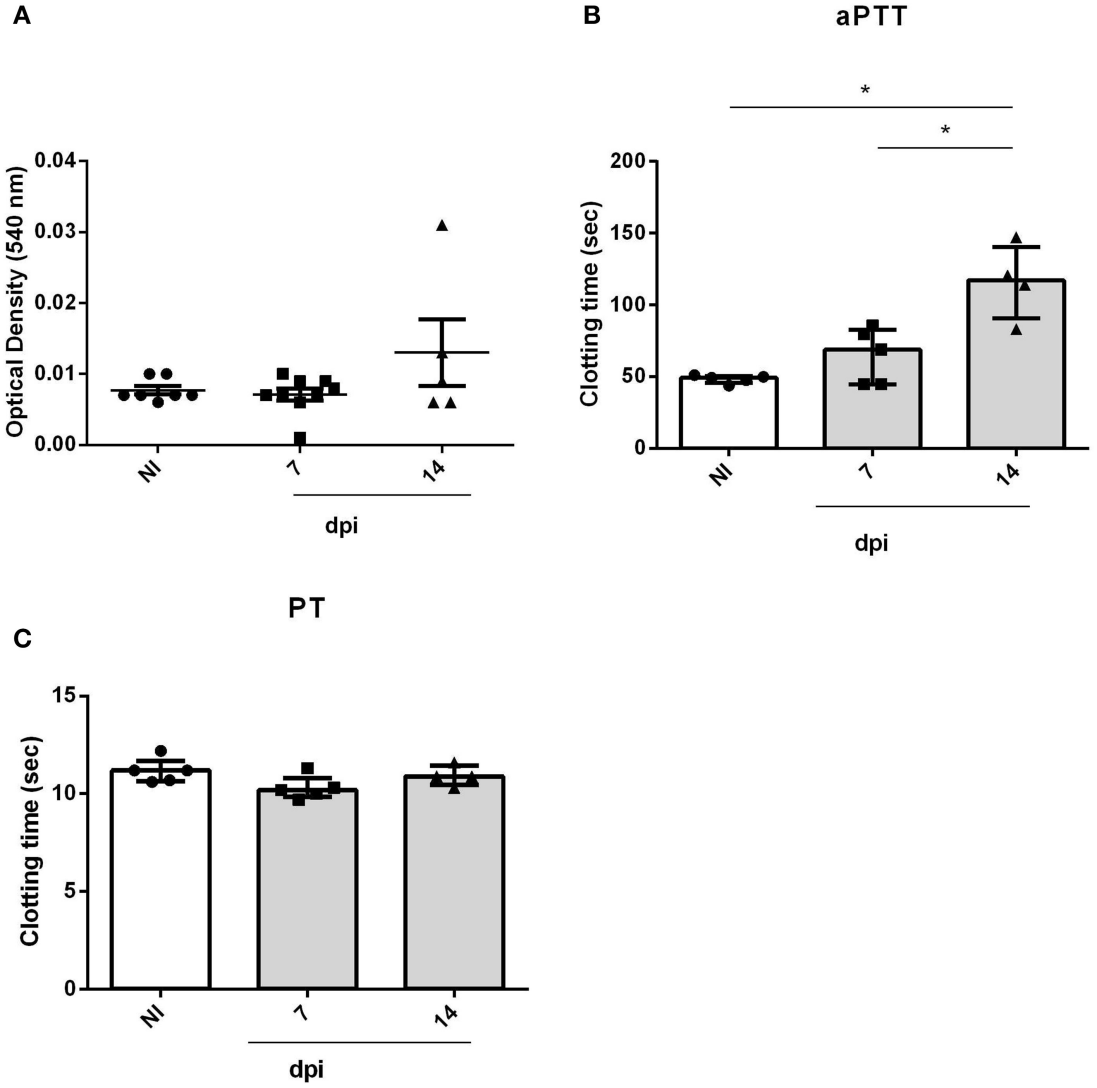
Bleeding from the tail and *ex vivo* PT and aPTT measurements of BALB/c mice during subcutaneous infection. Male BALB/c mice were subcutaneously infected with 5 × 10^4^ culture-derived *T. cruzi* trypomastigotes (Tulahuén strain). **(A)** Bleeding was caused by a tail transection in NI, 7 and 14 dpi SC mice. Absorbance at 540 nm (hemoglobin concentration) was used to estimate blood loss. **(B,C)** NI or SC plasma samples were obtained by cardiac puncture followed by addition of aPTT or PT reagent. Clotting time was estimated using a coagulometer. **(A-C)** Values are presented as mean ± SEM for each group/day post-infection and are representative of one experiment. Results were analyzed using one way ANOVA with Tukey's multiple comparisons test (*0.0001 < *p* < 0.05).

Orally infected mice showed hemostatic alterations in the acute phase. However, we wondered whether these changes were related to the oral transmission or with *T. cruzi* presence in the bloodstream. To answer this point, mice were infected with the same inoculum by the SC route. As demonstrated in Figure 5, SC infected mice also showed a significant increase in aPTT (Figure 5B) but not in bleeding tendency or PT (Figures 5A,C). Unlike OI mice, changes in aPTT of SC animals started at 14 dpi, prior to changes in OI animals (21 dpi).

At the SC route, infection killed all animals between 15 and 16 dpi.

## Discussion

Previous studies indicate a relationship between inflammation and coagulation in infection (20, 21). Some patients with oral ACD present symptoms related to hemostatic alterations such as facial and lower limbs edema, minor bleeding manifestations, most commonly from nose, skin petechiae or bruising and risk of thromboembolism (7, 11–13) indicating the need of exploring the crosstalk between immune and hemostatic systems. In the present study, we examined if the systemic inflammation led to hemostatic abnormalities in oral ACD and how blocking of IL-6 signaling pathway can modulate these changes.

We found that OI mice had significantly higher TNF, IL-6, and IFN-γ circulating levels than controls, thus demonstrating that *T. cruzi* infection leads to a potent pro-inflammatory systemic response. This is consistent with previous data showing that Tulahuén strain of *T. cruzi* induces TNF and IFN-γ production in BALB/c and C57BL/6 following subcutaneous (32) or OI (16). Moreover, high levels of TNF are involved in the toxic shock seen in IL-10-deficient mice infected intraperitoneally with 50 blood trypomastigotes of the Tulahuén strain of *T. cruzi* (33) as well as in cardiac, hepatic and spleen injury (16, 17). IFN-γ and IL-6 control parasite multiplication and confers host resistance (18, 34, 35). Furthermore, chronic patients with Chagas disease have elevated circulating levels of IL-6 when compared to healthy individuals (29, 36).

Interestingly, proinflammatory cytokines play a central role in the differential effects upon the coagulation and fibrinolysis pathways. TF is strongly induced after inflammatory stimuli mainly on monocytes and endothelial cells (23). Cytokines that have the ability to increase TF expression are TNF, IL-1β, IL-6, IFN-γ and the chemokine CCL2 (19, 37). Injection of low doses of LPS in healthy volunteers induced endotoxemia and TF mRNA had a 125-fold increase in whole blood cells (38). Blocking IL-6 with a monoclonal antibody in a primate model of sepsis, largely prevented LPS-induced coagulation activation once decreased significantly levels of prothrombin fragment 1+2 (F1+2) and thrombin-antithrombin complex (26). Contrarily, the same treatment in humans did not reduce LPS-induced TF mRNA or plasma concentrations of the same markers of coagulation activation showing that results obtained from primates may not automatically be transferable into humans. IL-6 is also involved in platelet thrombogenicity, once after addition of this cytokine to whole blood samples of healthy individuals, a marked spreading and clumping of the platelets was induced indicating an hyper-activation state (39).

Although the coagulation alterations mentioned above have been demonstrated to occur *in vivo* as a general response to pro-inflammatory stimuli, it is likely that other hematological changes may occur. *Trypanosoma cruzi* infection in humans is associated with anemia, thrombocytopenia and leukocytosis, mainly during the acute phase of disease (1, 40). Studies in inoculated mice with different *T. cruzi* strains also revealed anemia and thrombocytopenia during the acute phase of infection (41, 42). The mechanisms underlying these changes in blood count are not fully understood, but bone marrow suppression in hemopoiesis may be involved (42). In a second vein, the parasite trans-sialidase may induce the accelerated clearance of the platelets after depleting their sialic acid content that could lead to the thrombocytopenia observed during ACD (43). In a review of 31 published studies concerning hematological alterations in non-human hosts infected with *T. cruzi* (44), half of the studies reported anemia. However, we did not find significant changes in the erythrogram. An explanation could be the different route of inoculation.

Additionally, increased numbers of leukocytes were observed on 21 and 28 dpi. This alteration is also described in patients and in animal models (44–47). At 21 dpi, when platelet counts were still significantly lower, the normal platelet plug formation was affected as ascertained by tail bleeding assay. Notwithstanding, thrombocytopenia in humans with functional platelets generally does not induce or induces only minor bleeding symptoms, with the exception of life-threatening hemorrhages (48, 49). By contrast, mice with severe thrombocytopenia and inflammation resulted in spontaneous hemorrhage in different organs (50). Also at 21 dpi, beyond reduction in platelet plug formation, coagulation changes were seen with increased aPTT. Interestingly, production of D-dimer was evaluated and was detected at 14 and 21 dpi and FVIII levels were very low at 14 dpi.

Taken together, the disturbances mentioned above characterize the clinical syndrome of disseminated intravascular coagulation (DIC). This syndrome corresponds to a derangement of hemostasis with hemorrhage being the most common presentation consisting of widespread production of thrombin, which in turn leads to microvascular thrombosis, organ failure, and a consumptive coagulopathy related to a systemic inflammation (19). In fact, we previously showed formation of thrombotic masses in the liver of OI mice (16). Patients with DIC have higher IL-6 levels with the cytokine increase being paralleled by the severity of the disease (51). Therefore, we hypothesized that blockade of the IL-6 signaling would protect or minimize the hematological disturbances observed at 21 dpi. Yet, there was an impressive reduction in inflammation associated changes comprising significant reduction in coagulation time, aPTT and bleeding time. Both did not differ from control levels. However, anti-TNF treatment did not change these parameters, suggesting that TNF is not affecting directly the coagulation cascade, as observed in sepsis (26, 27).

**Figure 6:**
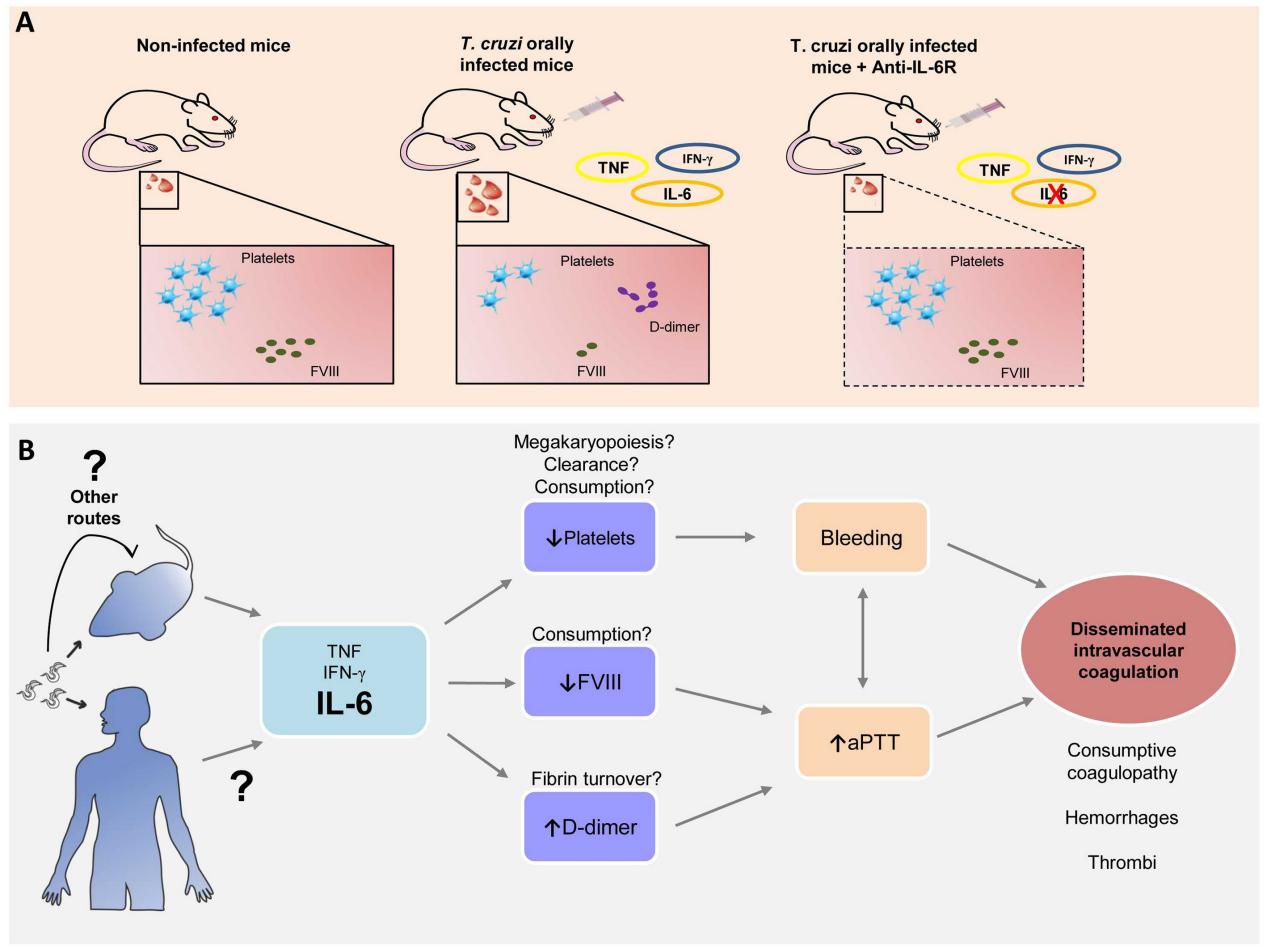
*Trypanosoma cruzi* oral infection causes hemostatic derangement linked to a systemic inflammation. **(A)** *Trypanosoma cruzi* orally infected mice have thrombocytopenia, Factor VIII depletion and increased D-dimer levels, linked to a systemic inflammation. Blocking of IL-6 signaling restores normal hemostasis. **(B)** Hypothesis: Acute *T. cruzi* infection leads to increase of pro-inflammatory cytokines. IL-6 is associated with a decrease with platelet count, possibly due to megakaryopoiesis disturbances, platelet clearance and/or consumption; reduction in serum FVIII levels and high levels of D-dimer related to fibrin turnover. Altogether these processes are involved in the increase of bleeding tendency and aPTT, signs of disseminated intravascular coagulation that leads to microhemorrhages and microthrombi as previously reported (7, 13, 16).

Thus, based on these early findings, targeting the immune system, more specifically IL-6R, during the acute phase of oral infection, can prevent a hemostatic derangement (Figure 6A). Since any long-term benefits and liabilities of the intervention still remain uncertain, it will be interesting to explore whether long-term benefits are also gained in our model of *T. cruzi* oral infection.

This study instigates many questions to explore. The hematological disturbances also develop in a parenteral route of transmission such as subcutaneously, suggesting that inflammatory response to parasite presence in the blood triggers this process. Moreover, whether there is an influence of the genetic variability of the parasite, i.e., *T. cruzi* DTUs (named as TcI to TcVI) correlated with more severe hematological changes. TcI, TcII, TcIII, TcIV, and TcVI genotypes had been reported in oral outbreaks (52–54) even though in the Brazilian Amazon prevails TcI (55) and in this study we used Tulahuén strain (TcVI). Ultimately, if there is any dysregulation of megakaryopoiesis leading to thrombocytopenia, changes in platelet clearance or consumption, deep alterations in fibrinolytic system remains undetermined (Figure 6B).

In conclusion, we show for the first time that oral ACD promotes a hemostatic derangement linked to systemic inflammation. This process is associated to low platelet count, bleeding and increased coagulation time, in parallel with high parasitemia. Blocking IL-6 signaling pathway ameliorates all these changes. Our studies open a new paradigm of looking to the hemostatic system when evaluating a patient infected with *T. cruzi* and suggest that translation of these results may be possible in the near future.

## Ethics Statement

This study was carried out in accordance with the recommendations of the Guide for the Care and Use of Laboratory Animals of the Brazilian National Council of Animal Experimentation and the Federal Law 11.794 (10/2008) Institutional Ethics Committee for Animal Research of the Oswaldo Cruz Foundation (CEUA-FIOCRUZ). The protocol was approved by CEUA-FIOCRUZ, License: L-028/16.

## Author Contributions

Conceived and designed the experiments: DA, RM, and JdM. Performed the experiments: DA, AM-D-S, MR, BM, CM, and DF-d-O. Analyzed the data: DA, WS, and JdM. Wrote the manuscript: DA, WS, RM, and JdM. All authors read and revised the manuscript.

### Conflict of Interest Statement

The authors declare that the research was conducted in the absence of any commercial or financial relationships that could be construed as a potential conflict of interest.
