# Supplementary material for: Oral Route Driven Acute Trypanosoma cruzi Infection Unravels an IL-6 Dependent Hemostatic Derangement
Source: Front Immunol. 2019 May 14;10:1073. doi: 10.3389/fimmu.2019.01073 (PMC6527737; doi:10.3389/fimmu.2019.01073)
Supplement: Supplementary file 1 [file Data_Sheet_1.PDF]

Figure S1 in Supplementary Material

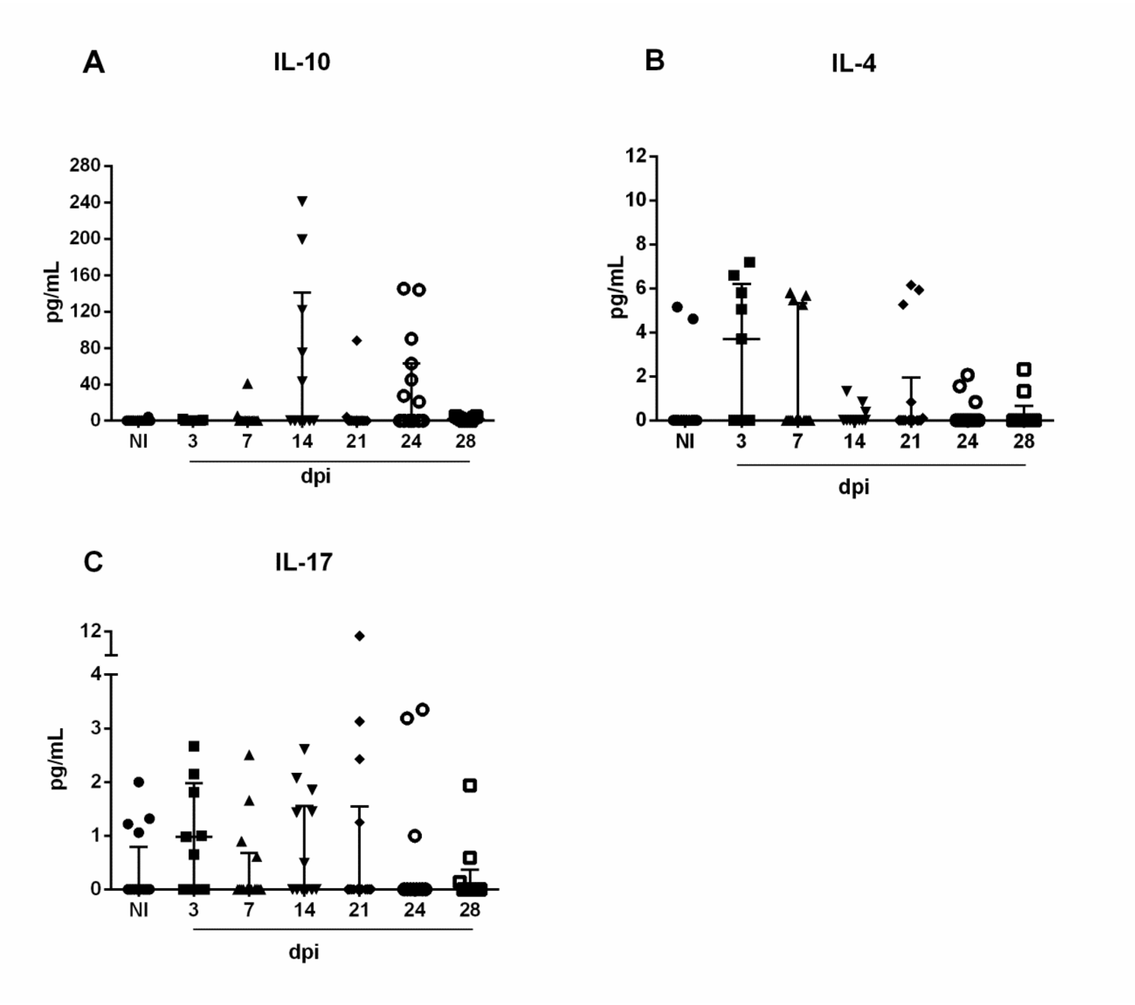

Fig S1. Th2 and Th17 serum cytokine levels during oral acute *T. cruzi* infection. Male BALB/c mice were infected with $5x10^4$ insect-derived metacyclic forms of *T. cruzi* within the oral cavity. In the course of the acute infection, serum was isolated and levels of IL-10 (A), Il-4 (B) and IL-17 (C) were quantified in non-infected (NI) and infected mice by the CBA method. Values represent the median with interquartile range for each group/day post-infection and are representative of two independent experiments. Results were analyzed using Kruskal-Wallis with Dunn's multiple comparisons test. Statistically significant differences among the groups were not detected.

Figure S2 in Supplementary Material

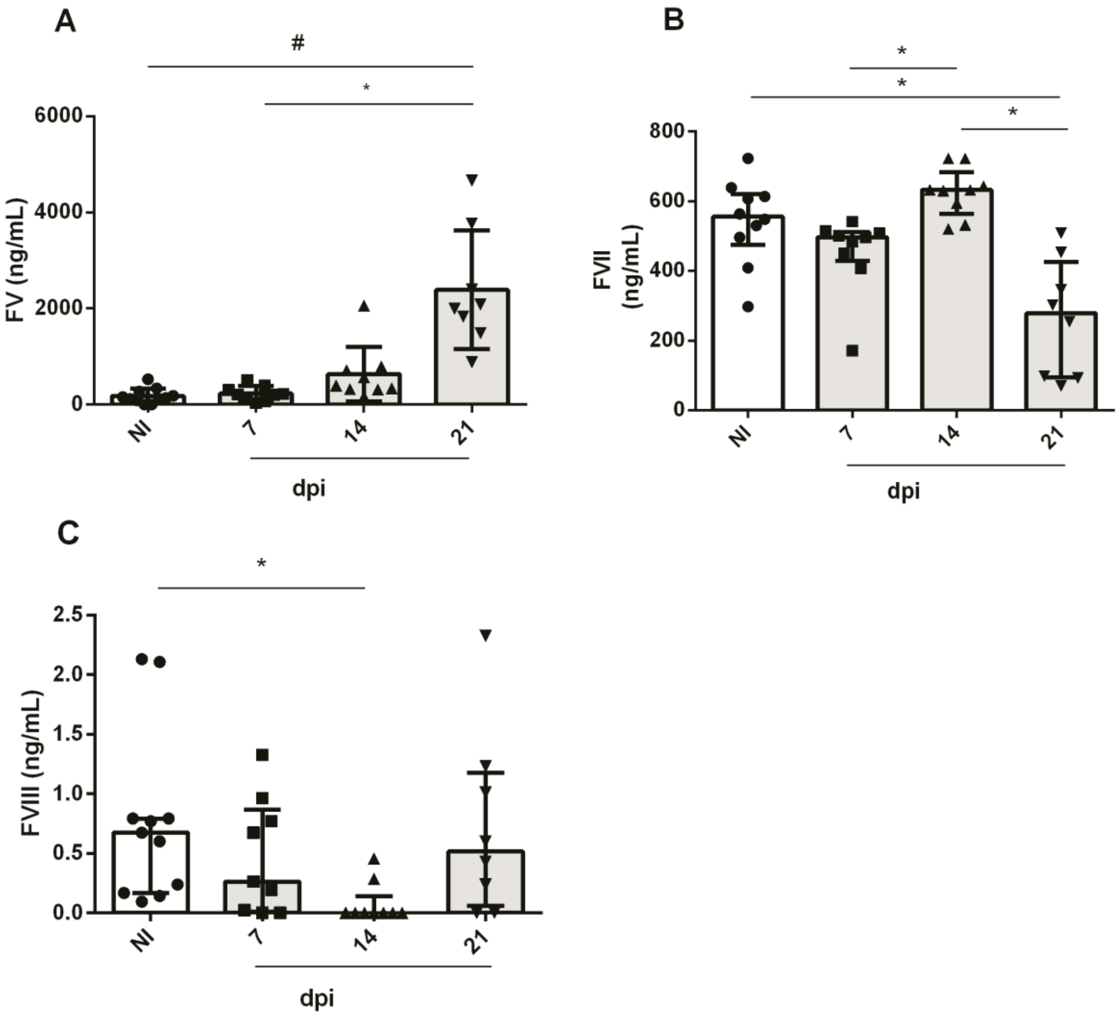

Fig S2. Changes in serum concentration of circulating coagulation factors during acute phase of oral *T. cruzi* infection. Male BALB/c mice were infected with $5x10^4$ insect-derived metacyclic forms of *T. cruzi* within oral cavity. NI or OI serum were obtained by cardiac puncture and used to measure levels of coagulation factors by ELISA. FV (A); FVII (B); FVIII (C). Values represent the median with interquartile range for each group/day post-infection and are representative of two independent experiments. Results were analyzed using Kruskal-Wallis with Dunn’s multiple comparisons test (* 0,0001<p<0,05, # p<0,0001).

Figure S3 in Supplementary Material

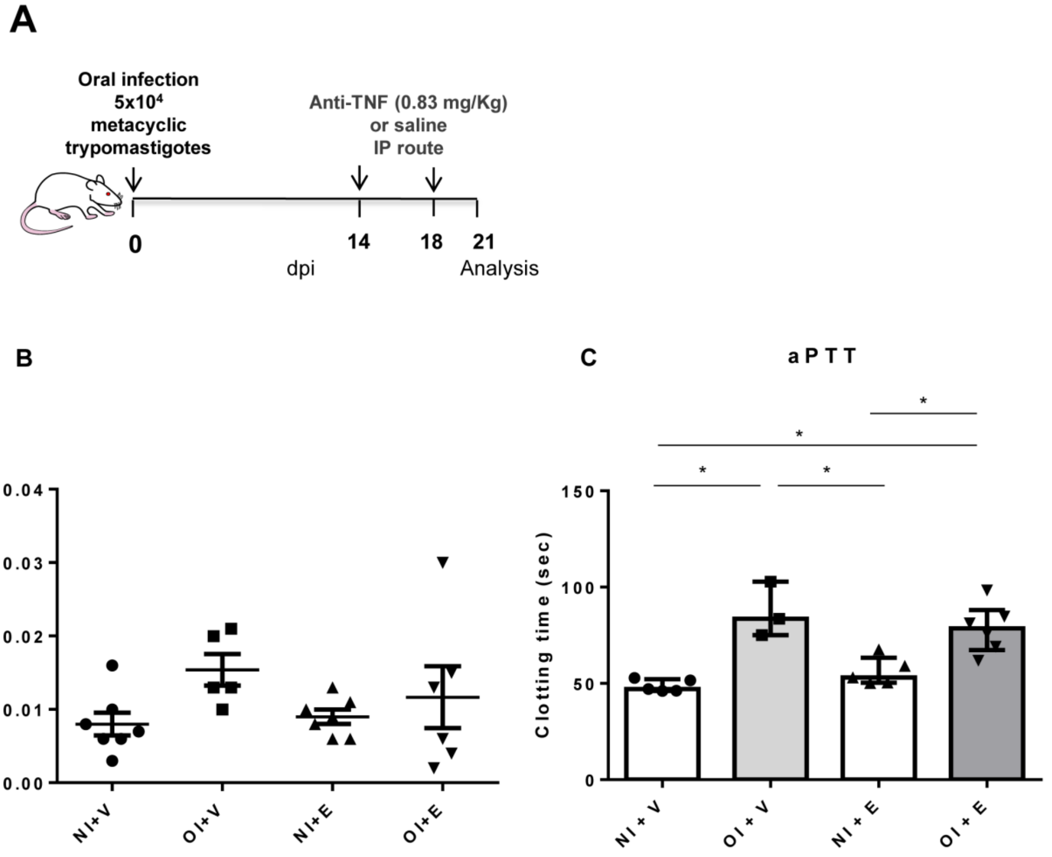

Fig S3. Coagulation test aPTT and tail bleeding assay in NI+V, OI+V, NI+E and OI+E mice. (A) Male BALB/c mice were infected with 5x10^{4} insect-derived metacyclic forms of *T. cruzi* (Tulahuén strain) within oral cavity. Enbrel® treatment began after 14 dpi and was performed again at 18 dpi. (B) Bleeding was caused by a tail transection in NI+Vehicle (NI+V), OI+V, NI+Enbrel® (NI+E) and OI+E. Absorbance at 540 nm (hemoglobin concentration) was used to estimate blood loss. (C) NI+V, OI+V, NI+E and OI+E plasma were obtained by cardiac puncture followed by addition of a aPTT reagent as described in the "Methods" section. Clotting time was estimated using a coagulometer. Values are presented as mean±SEM for each group/day post-infection and are representative of two independent experiments. Results were analyzed using one way ANOVA wit Tukey's multiple comparisons test (* 0,0001<p<0,05, # p<0,0001).
